# RETRACTION: **Biomechanical asymmetries persist after ACL reconstruction: Results of a 2‐year study**

**DOI:** 10.1002/jeo2.70189

**Published:** 2025-04-13

## Abstract

**RETRACTION:** F. Sharafoddin‐Shirazi, A. Letafatkar, J. Hogg, and V. Saatchian, “Biomechanical Asymmetries Persist After ACL Reconstruction: Results of a 2‐Year Study,” *Journal of Experimental Orthopaedics* 7, no. 1 (2019): 86, https://doi.org/10.1186/s40634-020-00301-2.

The above article, published online on 6 November 2020 in Wiley Online Library (wileyonlinelibrary.com), has been retracted by agreement between the journal Editor‐in‐Chief, Stefano Zaffagnini; European Society of Sports Traumatology, Knee Surgery and Arthroscopy; and John Wiley & Sons Ltd. The journal received concerns from a third party that detailed inconsistencies and unclear results in the published article. The authors responded to an inquiry by the publisher and supplied the original data. A review of the data by the journal confirmed that the statistical analyses contained errors that fundamentally impacted the data presented in the article and that several figures contained incorrect findings. The retraction has been agreed to because the errors in the data and figures fundamentally compromise the conclusions presented in this article. Author J. Hogg agrees with the retraction. Authors F. Sharafoddin‐Shirazi and A. Letafatkar disagree with the retraction. Author V. Saatchian did not respond to our notice regarding the retraction.

**RETRACTION:** F. Sharafoddin‐Shirazi, A. Letafatkar, J. Hogg, and V. Saatchian, “Biomechanical Asymmetries Persist After ACL Reconstruction: Results of a 2‐Year Study,” *Journal of Experimental Orthopaedics* 7, no. 1 (2019): 86, https://doi.org/10.1186/s40634-020-00301-2.

The above article, published online on 6 November 2020 in Wiley Online Library (wileyonlinelibrary.com), has been retracted by agreement between the journal Editor‐in‐Chief, Stefano Zaffagnini; European Society of Sports Traumatology, Knee Surgery and Arthroscopy; and John Wiley & Sons Ltd. The journal received concerns from a third party that detailed inconsistencies and unclear results in the published article. The authors responded to an inquiry by the publisher and supplied the original data. A review of the data by the journal confirmed that the statistical analyses contained errors that fundamentally impacted the data presented in the article and that several figures contained incorrect findings. The retraction has been agreed to because the errors in the data and figures fundamentally compromise the conclusions presented in this article. Author J. Hogg agrees with the retraction. Authors F. Sharafoddin‐Shirazi and A. Letafatkar disagree with the retraction. Author V. Saatchian did not respond to our notice regarding the retraction.

